# Spatial distribution of CD3- and CD8-positive lymphocytes as pretest for POLE wild-type in molecular subgroups of endometrial carcinoma

**DOI:** 10.3389/fmed.2023.1110529

**Published:** 2023-03-23

**Authors:** Samuel H. Jungen, Luca Noti, Lucine Christe, Jose A. Galvan, Inti Zlobec, Michael D. Müller, Sara Imboden, Franziska Siegenthaler, Joseph W. Carlson, Teijo Pellinen, Victoria Heredia-Soto, Ignacio Ruz-Caracuel, David Hardisson, Andres Redondo, Marta Mendiola, Tilman T. Rau

**Affiliations:** ^1^Institute of Pathology, University of Bern, Bern, Switzerland; ^2^Department of Obstetrics and Gynecology, University Hospital of Bern, University of Bern, Bern, Switzerland; ^3^Karolinska Institutet, Klinisk Patologi KS, Solna, Sweden; ^4^Keck School of Medicine of USC, Pathology, Health Sciences Campus, Los Angeles, CA, United States; ^5^Institute for Molecular Medicine Finland, Helsinki, Finland; ^6^Instituto de Investigación Biomédica del Hospital Universitario La Paz (IdiPAZ), Madrid, Spain; ^7^Centro de Investigación Biomédica en Red de Cáncer (CIBERONC), Instituto de Salud Carlos III, Madrid, Spain; ^8^Department of Pathology, Hospital Universitario La Paz, Madrid, Spain; ^9^Faculty of Medicine, Universidad Autónoma de Madrid, Madrid, Spain; ^10^Department of Medical Oncology, Hospital Universitario La Paz, Madrid, Spain; ^11^Institute of Pathology, Universitätsklinikum Düsseldorf, Düsseldorf, Germany

**Keywords:** endometrial carcinoma, TCGA, POLE, CD3, CD8, multiplex immunofluorescence

## Abstract

**Introduction:**

Over the years, the molecular classification of endometrial carcinoma has evolved significantly. Both POLEmut and MMRdef cases share tumor biological similarities like high tumor mutational burden and induce strong lymphatic reactions. While therefore use case scenarios for pretesting with tumor-infiltrating lymphocytes to replace molecular analysis did not show promising results, such testing may be warranted in cases where an inverse prediction, such as that of POLEwt, is being considered. For that reason we used a spatial digital pathology method to quantitatively examine CD3^+^ and CD8^+^ immune infiltrates in comparison to conventional histopathological parameters, prognostics and as potential pretest before molecular analysis.

**Methods:**

We applied a four-color multiplex immunofluorescence assay for pan-cytokeratin, CD3, CD8, and DAPI on 252 endometrial carcinomas as testing and compared it to further 213 cases as validation cohort from a similar multiplexing assay. We quantitatively assessed immune infiltrates in microscopic distances within the carcinoma, in a close distance of 50 microns, and in more distant areas.

**Results:**

Regarding prognostics, high CD3^+^ and CD8^+^ densities in intra-tumoral and close subregions pointed toward a favorable outcome. However, TCGA subtyping outperforms prognostication of CD3 and CD8 based parameters. Different CD3^+^ and CD8^+^ densities were significantly associated with the TCGA subgroups, but not consistently for histopathological parameter. In the testing cohort, intra-tumoral densities of less than 50 intra-tumoral CD8^+^ cells/mm^2^ were the most suitable parameter to assume a POLEwt, irrespective of an MMRdef, NSMP or p53abn background. An application to the validation cohort corroborates these findings with an overall sensitivity of 95.5%.

**Discussion:**

Molecular confirmation of POLEmut cases remains the gold standard. Even if CD3^+^ and CD8^+^ cell densities appeared less prognostic than TCGA, low intra-tumoral CD8^+^ values predict a POLE wild-type at substantial percentage rates, but not vice versa. This inverse correlation might be useful to increase pretest probabilities in consecutive POLE testing. Molecular subtyping is currently not conducted in one-third of cases deemed low-risk based on conventional clinical and histopathological parameters. However, this percentage could potentially be increased to two-thirds by excluding sequencing of predicted POLE wild-type cases, which could be determined through precise quantification of intra-tumoral CD8^+^ cells.

## Introduction

Risk stratification for endometrial carcinoma (EC) is based on morphological features and molecular findings proposed by The Cancer Genome Atlas (TCGA) (1, 2). These results were implemented into the 2021 guidelines of the European Society of Gynecological Oncology (ESGO) (3). In parallel, the diagnostic and prognostic influence of tumor infiltrating lymphocytes (TIL) in EC is intensively investigated (4–9). However, it is not yet possible to stage patients with ECs based on an immune infiltrate like that proposed by the Immunoscore for colorectal cancer (10–12).

Endometrial cancers are divided into four molecular categories: (I) polymerase epsilon mutated (POLEmut), (II) mismatch repair-deficient (MMRdef), (III) a type with no specific mutations (NSMP) or p53 wild-type, and (IV) cases with p53 mutations (p53abn) (2).

Among them, ECs POLE mutated have the best prognosis (13, 14). It is proposed that a high tumor mutational burden leads to strong responses of TILs and to a better clinical outcome (6, 9, 14). POLEmut tumors are associated with high counts of intra-tumoral CD3^+^ and CD8^+^ immune cells and an enhanced cytotoxic reaction (5–8). Such tumors also possess high counts of PD1 on TILs, counterbalancing the strong immune reaction with this inhibitory protein (9, 13). Due to their excellent prognosis, FIGO stage I and II POLEmut tumors are not selected for checkpoint inhibition therapy (3, 6). Therefore, detection of POLEmut tumors is mainly linked to de-escalating therapeutic strategies.

Unfortunately, POLEmut tumors are rare (∼10%) and cannot be sorted on traditional histopathological features; therefore, sequencing is necessary (3, 14, 15). We hypothesize that many POLE wild-type cases could be ruled out by a detailed analysis of spatial patterns of the immune infiltrate. Multiplex immunofluorescence as our method of choice accurately quantifies CD3^+^ and CD8^+^ cells in specific regions of the tumor microenvironment and is superior to HE-based TIL evaluation or single-target immunohistochemistries (3, 15, 16).

We combined this approach with molecular subtypes classification and spatial analysis to investigate the diagnostic and prognostic impact of CD3^+^ and CD8^+^ lymphocytes in EC (17). Finally, the plethora of data was shrunk to an applicable diagnostic pretest based on the immune infiltrate to detect and exclude POLE wild-type cases before sequencing.

## Materials and methods

### Patient cohorts

A detailed description of the patient cohort was published previously (17). A total of 252 patient tumor samples with EC were collected retrospectively at the University Hospital Inselspital (Bern, Switzerland) and served as the testing cohort. All samples were reevaluated with the use of the 4th edition of the WHO classification and the 8th edition of the TNM classification (Table 1) (18, 19).

**TABLE 1:** Patient characteristics.

Feature	Characteristics	Testing cohort freq N (%)	Validation cohort freq N (%)	Total freq N (%)
Patient age		< 65: 108 (42.9%)	<65: 102 (47.9%)	< 65: 210 (45.2%)
		> 65: 144 (57.1%)	> 65: 111 (52.1%)	> 65: 255 (54.8%)
Histological subtype	Endometrioid	239 (94.8%)	186 (87.3%)	425 (91.4%)
	non-endometrioid:	13 (5.2%)	27 (12.7%)	40 (8.6%)
	serous	7 (2.8%)	16 (7.5%)	23 (4.9%)
	clear cell	2 (0.8%)	2 (0.9%)	4 (0.9%)
	undifferentiated	1 (0.4%)	6 (2.9%)	7 (1.5%)
	mixed	3 (1.2%)	3 (1.4%)	6 (1.3%)
T-category	pT1a	121 (48.0%)	148 (69.5%)	269 (57.8%)
	pT1b	70 (27.7%)	53 (24.9%)	123 (26.5%)
	pT2	33 (13.1%)	12 (5.6%)	45 (9.9%)
	pT3a	12 (4.8%)	0 (0.0%)	12 (2.6%)
	pT3b	13 (5.2%)	0 (0.0%)	13 (2.8%)
	pT4	2 (0.8%)	0 (0.0%)	2 (0.4%)
N-category	cN0	50 (19.8%)	0 (0.0%)	50 (10.8%)
	cN1	1 (0.4%)	0 (0.0%)	1 (0.2%)
	pN0	159 (63.1%)	213 (100.0%)	372 (80.0%)
	pN1	42 (16.7%)	0 (0.0%)	42 (9.0%)
Tumor grade	unknown	0 (0.0%)	25 (11.7%)	25 (5.4%)
	G1	91 (36.1%)	123 (57.7%)	214 (46.0%)
	G2	107 (42.5%)	43 (20.2%)	150 (32.3%)
	G3	54 (21.4%)	22 (10.4%)	76 (16.3%)
Lymphatic invasion	L0	196 (77.8%)	170 (79.8%)	366 (78.7%)
	L1	56 (22.2%)	43 (20.2%)	99 (21.3%)
Venous invasion	V0	198 (78.6%)	203 (95.3%)	401 (86.2%)
	V1	54 (21.4%)	10 (4.7%)	64 (13.8%)
MELF pattern	Unknown	0 (0.0%)	32 (15.0%)	32 (6.9%)
	Present	48 (19.0%)	23 (10.8%)	71 (15.3%)
	Not present	204 (81.0%)	158 (74.2%)	362 (77.8%)
TCGA subgroups	POLE mut.	10 (4.0%)	12 (5.6%)	22 (4.7%)
	MMR def.	80 (31.7%)	65 (30.5%)	145 (31.2%)
	NSMP	130 (51.6%)	108 (50.7%)	238 (51.2%)
	p53 abn.	32 (12.7%)	28 (13.2%)	60 (12.9%)
Total		n = 252	n = 213	n = 465

An additional 213 patients with early stage EC from the Hospital Universitario La Paz (Madrid, Spain) were included as the validation cohort (Table 1). The detailed description of the characteristics of the validation cohort was published previously (20).

### Tissue microarray construction

**FIGURE 1:**
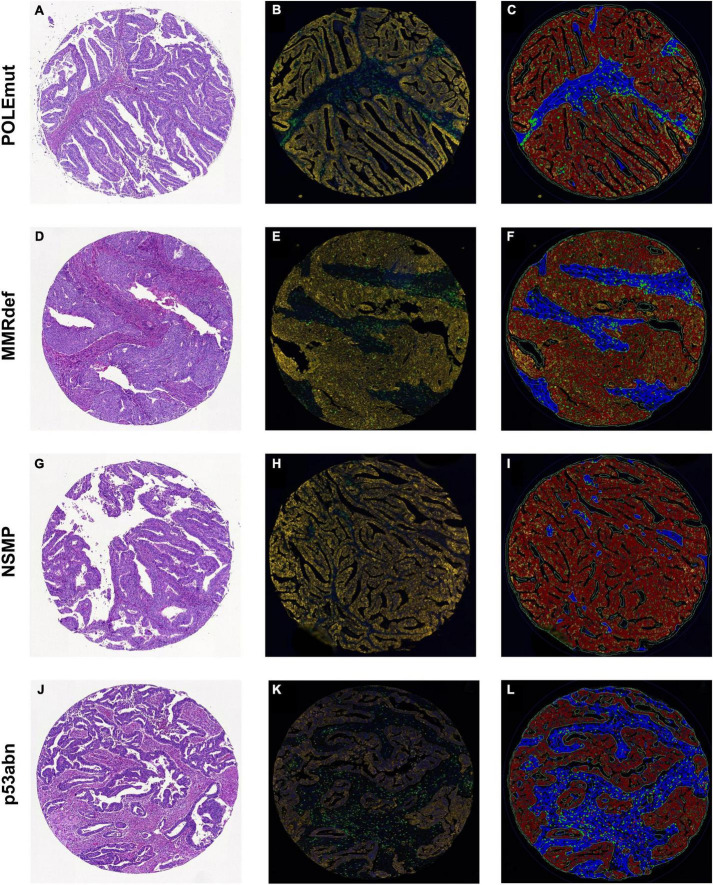
Sample cores for every subgroup of The Cancer Genome Atlas. **(A,D,G,J)** Hematoxylin and eosin. **(B,E,H,K)** Multiplexed immunofluorescence. **(C,F,I,L)** After digital image analysis. **(A–C)** POLEmut. **(D–F)** MMRdef. **(G–I)** NSMP. **(J–L)** p53abn. Note the gradient between POLE mutated tumors with highest infiltrate of both CD3^+^ (green) and CD8^+^ (red) lymphocytes to NSMP tumors with lowest CD3^+^ and CD8^+^ cells, with MMRdef and p53abn tumors in between.

For the testing cohort, a next-generation tissue microarray (ngTMA^®^) was constructed using principles published previously (17, 21, 22). Punch biopsies with a diameter of 1.5 mm were collected in triplicate from the tumor center and invasive front (Figure 1 and Table 2).

**TABLE 2:** Construction features of testing cohort TMAs.

	Valid core numbers		≪Intratumoral≫ area/core		≪Close≫`area/core		≪Distant≫ area/core		≪Total≫ area/core	
Tumor center	566	74%	1.25 mm^2^	61%	0.50 mm^2^	24%	0.32 mm^2^	15%	2.07 mm^2^	100%
Invasive front	589	77%	0.66 mm^2^	37%	0.36 mm^2^	20%	0.77 mm^2^	43%	1.79 mm^2^	100%

To avoid overestimation of densities detailed areas were excluded if less than 0.01mm^2^ could be obtained.

For the validation cohort, tissue microarrays were made with selected central tumor areas based on hematoxylin and eosin evaluation. 1.2 mm tissue punches were arranged in duplicate using a TMA workstation (Beecher Instruments, Silve Spring, MD, USA) (20).

### Multiplexed immunohistochemistry

**FIGURE 2:**
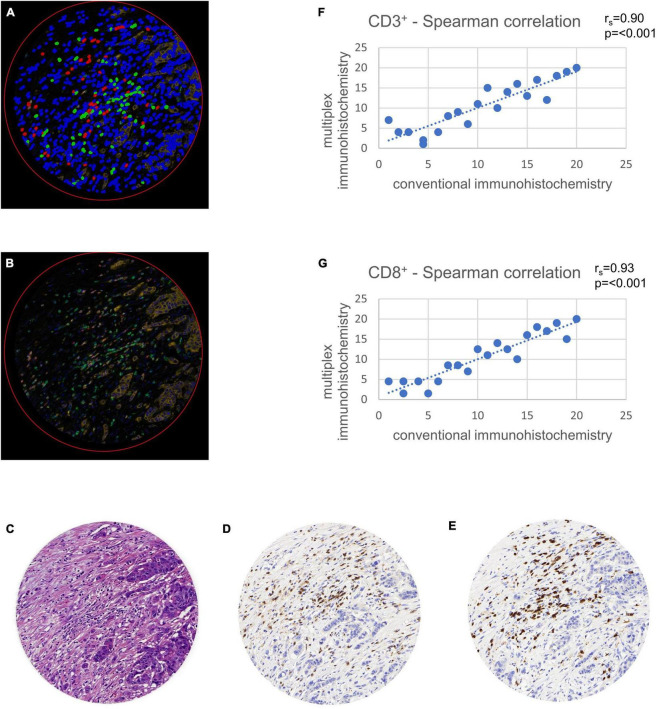
Validation of multiplex immunohistochemistry with conventional immunohistochemistry using tissue microarray (TMA) cores. TMAs were virtually set in a whole slide image of a colon cancer case stained in duplicate and were used for comparison of multiplex with conventional immunohistochemistry. Comparison was calculated with a Spearman correlation (r_*s*_). **(A)** Multiplexed immunofluorescence after digital image analysis with stromal cells (blue), CD3^+^ lymphocytes (green) and CD8^+^ lymphocytes (red). Note the possibility of multiplexed immunohistochemistry to stain double positive CD3^+^ and CD8^+^ cells in a single TMA core, leading to a more exact quantification of these cells. **(B)** Multiplexed immunofluorescence original data. **(C)** Hematoxylin and eosin staining. **(D)** Hematoxylin and diaminobenzidine staining showing CD3^+^ lymphocytes. **(E)** Hematoxylin and diaminobenzidine staining showing CD8^+^ lymphocytes. **(F)** Spearman correlation of CD3^+^ cell counts with conventional immunohistochemistry on *x*-axis and multiplex immunofluorescence on *y*-axis. **(G)** Spearman correlation of CD8^+^ cell counts with conventional immunohistochemistry on *x*-axis and multiplex immunofluorescence on *y*-axis.

For the testing cohort, an Opal Kit with four fluorescence colors (Akoya Biosciences, MA, USA) was used to stain the slides (16, 23–30). Cell counts of each fluorescence channel were validated with conventional stained slides (Figure 2). The fluorescence staining was executed following routine protocols on a Leica Bond RX autostainer (Leica Biosystems, Nussloch, Germany). Four antigens were detected with different colors to display nuclei, CD3^+^ and CD8^+^ lymphocytes, and tumor cells (Table 3). For analysis, CD3 positive T-cells were defined irrespective of CD8 status, whereas the latter was defined as a subgroup. Tumor cells were defined as PanCK positive, stromal cells as negative for CD3, CD8 and PanCK.

**TABLE 3:** Staining material for multiplexed immunofluorescence.

Antigen	Primary antibody	Species	Secondary antibody	Dilution	Wavelength	Color	Filter
**Testing cohort**							
Nuclei			HRP		Spectral DAPI	Blue	DAPI
CD3	Novocastra, NCL-L-CD3-56	mouse	HRP	1:200	Opal 520	Green	FITC
CD8	Dako, M710301	mouse	HRP	1:200	Opal 570	Red	TRITC
PanCK	Dako, M0821	mouse	HRP	1:400	Opal 620	Orange	CY5
**Validation cohort**							
Nuclei			HRP		Spectral DAPI	Blue	DAPI
CD3	Thermo, MA5-14482*	rabbit	HRP	1:300	TSA-555 Alexa-750	Red	
CD8	Dako, M7103*	mouse	HRP	1:300	Alexa-647	Green	
PanCK	panEpi-cocktail* Abcam, ab7753 Invitrogen, MA5-13156 BD, 610182	mouse	HRP	1:150 1:100 1:200	Alexa-750	Violet	

*Panel 1 and Panel 2.

The validation cohort was stained using two antibody panels and two staining cycles (Table 3). In both staining rounds, antibodies were amplified using Alexa fluorophores and tyramide signal amplification, counter-staining was done with 4,6-diamidino-2-phenylindole (DAPI). A detailed description of the staining process can be found here (16, 20).

### Scanning

All slides of the testing cohort were digitized in 8-bit with a Pannoramic 250 Flash II slide scanner (3D Histech, Budapest, Hungary) with fluorescence mode (Table 3). The scanned slides were imported in MRXS file format to the Case Viewer software (3D Histech, Budapest, Hungary) and exported as TILED TIFFs for further analysis.

For the validation cohort, TMAs were imaged as whole slides using a Zeiss Axio Scan.Z1 scanner (Zeiss Group, Oberkochen, Germany) with a Hamamatsu ORCA-Flash 4.0 V2 Digital CMOS Camera in 16-bit (Hamamatsu Corporation, Bridgewater, NJ, USA). The images were exported as Big TIFF with original raw channel data of four channels (DAPI, CD3, CD8 and PanCK) (20). After scanning, the images were merged into 4-channel RGB TIFFs and exported for further analysis.

### Digital image analysis

**FIGURE 3:**
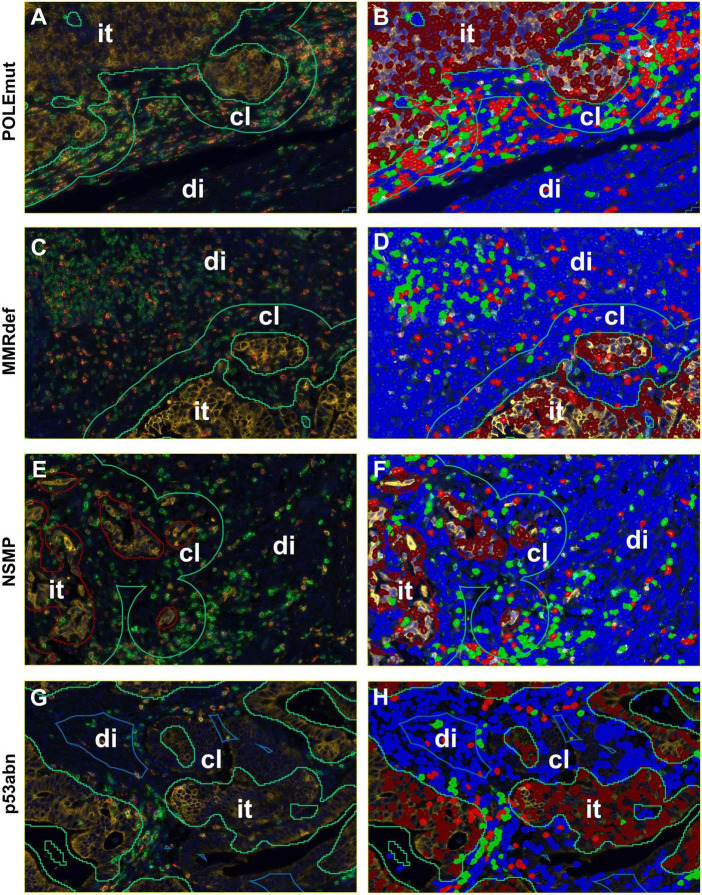
Detailed screenshots of regions. It = intra-tumoral, cl = close (< 50 microns away from tumor), di = distant (> 50 microns away from tumor). Blue = stroma cells, brown = tumor cells, green = CD3^+^ immune cells, and red = CD8^+^ immune cells. **(A,C,E,G)** Multiplexed immunofluorescence picture. **(B,D,F,H)** After digital image analysis. **(A,B)** POLEmut. **(C,D)** MMRdef. **(E,F)** NSMP. **(G,H)** p53abn. Note the different gradients between intra-tumoral, close, and distant regions concerning CD3^+^ and CD8^+^ lymphocyte counts according to the molecular subtypes indicated at the left.

Digital image analysis was executed with QuPath as open source tool (31, 32). Applying a script (Supplementary material), the TMAs were analyzed for cell count, percentage, and density of CD3^+^ and CD8^+^ lymphocytes in three different compartments (33). Tissue was detected with the ≪Pixel classifier≫ and defined as intra-tumoral, tumor neighborhood (close area of 50 microns), and tumor distant (Figures 1, 3 and Table 4) (34–36). The cut-off of 50 microns for a close to tumor region was taken from literature and appeared to be appropriate for TMA based analysis to allow appropriate tumor distant areas at core sizes of 1.2-1.5 mm (37). Cells were detected using the ≪Watershed cell detection≫ function. Appropriate thresholds for lymphocytes and tumor cells were calculated mathematically using the ≪Auto threshold≫ function of Fiji ImageJ (open source, GNU General Public License) with mean and maximum entropy models (Table 4) (16, 38). A minimum size of 0.01 mm^2^ per ROI was set as threshold to avoid overestimation of cell densities due to area as denominator. Per case, the mean of cell densities of each ROI was calculated from multiple corresponding cores within the TMAs. Regarding the validation cohort, mean and maximum threshold levels had to be adapted to the different staining intensities and to the spectrum of 16-bit images (Table 4). The identical automated workflow as described above was used to detect and count CD3^+^ and CD8^+^ lymphocytes in each compartment.

**TABLE 4:** Definition of variables.

Threshold	Nuclei	CD3^+^	CD8^+^	PanCK
**Testing cohort (8-bit, values 0-256)**				
Nucleus: mean	> = 35	> = 35	> = 35	
Cytoplasm: mean		> = 36	> = 33	> = 61
Cytoplasm: max		> = 96	> = 116	
**Validation cohort (16-bit, values 0-65535)**				
Nucleus: mean	> = 2364	> = 2364	> = 2364	
Cytoplasm: mean		> = 1968	> = 1213	
Cytoplasm: max		> = 5595	> = 9161	

### Troubleshooting

Auto-fluorescence from erythrocytes and tumor cells in FITC and CY5 channels was solved by applying basic anatomical principles to the script such as lymphocyte size and density of chromatin to define CD3^+^ and CD8^+^ lymphocytes. This allowed higher thresholds for lymphocyte signals compared to those of erythrocytes and tumor cells (Table 4).

Auto-fluorescent erythrocytes of the validation cohort were priorly removed using Ilastik-1.3.3post2 machine learning software (open source, GNU General Public License). The software was trained to differentiate between empty tissue, red blood cells, and “good” tissue with automatic removal of empty tissue and erythrocytes (20).

### POLE mutational status according to TCGA subtypes

POLE mutational status of both cohorts was detected by hotspot Sanger sequencing of the catalytic region of POLE covered in exons 9, 12 (testing cohort), 13, and 14 as previously published (17, 20) and under consideration of the POLE risk score for variant classification (39). Only consensus pathogenic POLE mutations entered the analysis. Both cohorts were further classified into separate subgroups performing immunohistochemistries for MLH1, MSH2, MSH6, PMS2, and p53 proteins as described before (17, 20).

### Statistics

Statistical analysis of both cohorts comprised descriptive statistics, Spearman correlations and Chi-square tests. As cell counts did not appear as normally distributed in Kolmogorov-Smirnow and Shapiro-Wilks test, Kruskal-Wallis-test, Mann-Whitney-U-tests and non-parametric median comparison were applied. If multiple comparisons were analyzed with Kruskal-Wallis-test the significances were adapted with Bonferroni correction. ROC curve analysis was used to determine cut-offs for diagnostic accuracy for molecular subtypes. Cut-offs for survival analysis were initially based on medians of averaged CD3^+^ and CD8^+^ densities and furthermore determined with the final cut-off. Results were outlined with log-rank tests and Kaplan-Meier curves. The threshold for statistical significance was set at three different layers marked with asterixes *p ≤ 0.05, ^**^p ≤ 0.01 and ^***^p ≤ 0.001. All calculations were performed with SPSS, version 29.

## Results

### Validation of the multiplexed immunohistochemistry approach

CD3 and CD8 for conventional DAB-based brightfield IHCs were previously standardized during participation in the Immunoscore project in colorectal cancer at the Institute of Pathology Bern (12). In a first step, we ensured an accurate transfer to an immunofluorescence approach on the testing cohort. The Opal method showed high correlations of lymphocyte cell counts in comparison with conventional immunohistochemistry. A Spearman correlation analysis comparing the two different staining methods showed significant concordance with r_*s*_ = 0.90 for CD3^+^ (*p* < 0.001) and r_*s*_ = 0.93 for CD8^+^ (*p* < 0.001) (Figure 2).

Furthermore, 99.81% of CD8-positive cells were also CD3-positive (CD3^+^CD8^+^). The remaining 0.19% of CD3-negative CD8-positive cells (CD3^–^CD8^+^) occured possibly to steric inhibition in multiplexing, but entered our analysis as CD8 positive. Hence, CD8^+^ represents almost completely a matured subgroup of cytotoxic T-cells of the general CD3^+^ population.

### Comparison of CD3^+^ and CD8^+^ densities with conventional clinical-pathological parameters in the testing cohort

Next, we compared CD3^+^ and CD8^+^ cell counts per mm^2^ with histological subtype, T-category, N-category, grading, lymphovascular invasion, hemangioinvasion, age and MELF pattern within the testing cohort. As mentioned above, cores were evaluated in total and the three detailed regions defined as intra-tumoral, close and distant.

In comparison of endometrioid versus non-endometrioid tumors, the testing cohort showed throughout higher lymphocytic counts in non-endometrioid tumors. However, differences in median cell densities for CD3^+^ did not reach significance. Regarding CD8^+^, these differences were evident as CD8^+^ in total areas of endometrioid subtype appeared with median values of 50/mm^2^ versus 121/mm^2^ in non-endometrioid (Mann-Whitney-U-test, *p* = 0.029). In tendency, this accounted for close areas (61/mm^2^ versus 205/mm^2^; Mann-Whitney-U-test, *p* = 0.069) and distant areas with median values of 51/mm^2^ versus 305/mm^2^ (Mann-Whitney-U-test, *p* = 0.036), as well.

Within the T-category, CD3^+^ and CD8^+^ counts did not show relevant differences. Only in CD8^+^ median densities of distant areas a comparison between pT1a and pT3 cases showed an increase from 39/mm^2^ to 92/mm^2^ (Kruskal-Wallis-test all T-categories *p* < 0.001 and pairwise median test, *p* = 0.004).

Within cases with nodal metastasis CD3^+^ densities showed no differences. Elevated medians in CD8^+^ densities were found in tendency in intra-tumoral (*p* = 0.087, Mann-Whitney-U-test) and significantly in close areas (*p* = 0.008, Mann-Whitney-U-test) with median values of 62/mm^2^ and 94/mm^2^ in comparison to the corresponding median values of 46/mm^2^ and 51/mm^2^ of the pN0 group, respectively.

In the testing cohort, grading was paralleled with stepwise higher CD3^+^ cell densities, with median values of 137/mm^2^ in G1, 153/mm^2^ in G2 and 196/mm^2^ in G3 tumors (Kruskal-Wallis-Test, *p* = 0.036), particularly pronounced in distant areas with medians of 140/mm^2^ in G1, 178/mm^2^ in G2 and 259/mm^2^ in G3 tumors (Kruskal-Wallis-Test, *p* = 0.011). For CD8^+^, advanced grading presented with total cell densities of 39/mm^2^ in G1, 96/mm^2^ in G2 and 209/mm^2^ in G3 tumors (Kruskal-Wallis-Test, *p* < 0.001), which could be split into differences from 46/mm^2^ to 75/mm^2^ to 100/mm^2^ in close areas (Kruskal-Wallis-Test, *p* < 0.001), as well as differences from 38/mm^2^ to 60/mm^2^ to 96/mm^2^ in distant areas (Kruskal-Wallis-Test, *p* < 0.001), respectively.

Lymphovascular invasion showed significantly higher CD8^+^ cell densities in close and distant areas, with medians of 92/mm^2^ and 89/mm^2^ for lymphovascular invasion versus 53/mm^2^ and 46/mm^2^ 143/mm^2^ for L0 cases (Mann-Whitney-U-Test *p* = 0.031 and *p* < 0.001, respectively). In hemangioinvasion similar differences were found in cell densities in distant areas with CD3^+^ values of 260/mm^2^ (V1) to 166^2^ (V0) and CD8^+^ values of 94/mm^2^ (V1) to 45/mm^2^ (V0) (Mann-Whitney-U-Test *p* = 0.004 and *p* < 0.001, respectively).

No significant dependencies were found between the CD3^+^ and CD8^+^ immune infiltrate and age (Spearman correlation) or MELF pattern (Kruskal-Wallis-Test).

**FIGURE 4:**
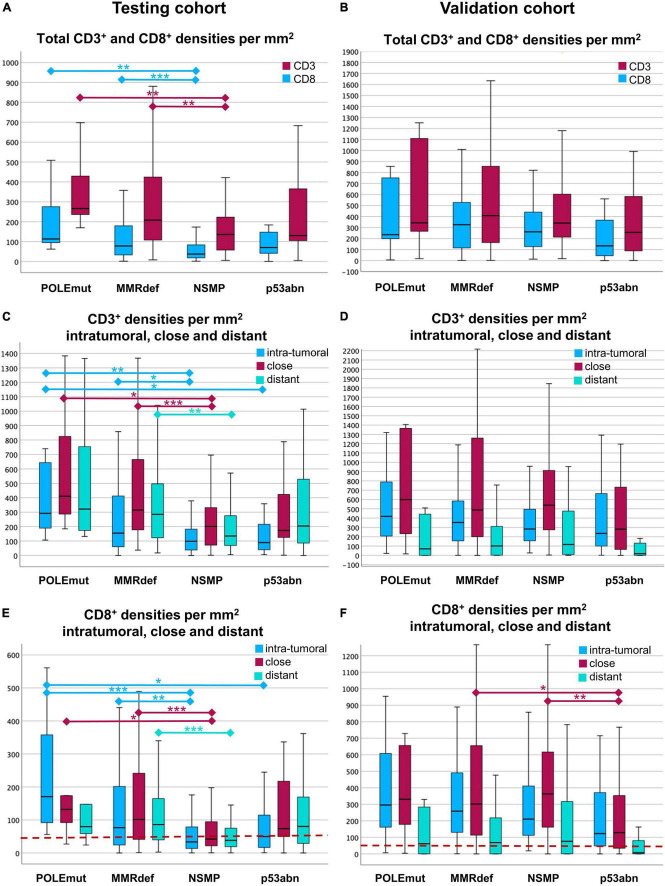
Boxplots of cell densities for the testing cohort **(A,C,E)** and validation cohort **(B,D,F)**. **(A,B)** Total cell densities per mm^2^ for CD3^+^ (red) and CD8^+^ (blue) cells in comparison with the four molecular subgroups according to The Cancer Genome Atlas (TCGA). POLE mutated tumors show the highest immune infiltrate, where NSMP and p53abn tumors show the lowest. **(C,D)** CD3^+^ cell densities per mm^2^ in the three compartments of a core. The compartments are intra-tumoral (blue), close (red), and distant (green). Note the gradient between the highest values for close and the lowest values for intra-tumoral CD3^+^ lymphocytes. **(E,F)** CD8^+^ cell densities per mm^2^ intra-tumoral (blue), close (red), and distant (green) for every TCGA group. Note the distinct gradients between intra-tumoral, close, and distant regions. In the testing cohort **(E)**, CD8^+^ lymphocytes are highest intra-tumoral in POLE mutated tumors. In the validation cohort **(F)**, the gradients are different to the testing cohort with highest values close to the tumor, except in p53abn tumors. The red line indicates the cut-off of 50 intra-tumoral CD8^+^ cells per mm^2^. Significances are outlined as non-parametric median comparison following Kruskal-Wallis Test marked with asterixes as *p ≤ 0.05, **p ≤ 0.01 and ***p ≤ 0.001.

As expected, the differences of CD3^+^ and CD8^+^ cell densities and TCGA groups were most pronounced (Kruskal-Wallis-Test, *p* < 0.001). In the testing cohort, the highest medians for CD3^+^ and CD8^+^ cell densities in total areas were found in POLE mutated tumors, followed by those with MMRdef. The lowest values were found in NSMP tumors, with p53 mutated tumors in between (Figure 4A).

Comparison of the three compartments showed the highest medians of CD3^+^ cell densities in the vicinity of the tumor, followed by areas distant from the tumor, and the lowest values were found for intra-tumoral areas. These findings were consistent for three TCGA groups, except for p53abn tumors (Kruskal-Wallis-Test, *p* < 0.001) (Figure 4C).

CD8^+^ cells behaved similarly (Kruskal-Wallis-Test, *p* < 0.001). Only with regards to the highest cell densities, the intra-tumoral region in POLEmut cases was more pronounced. Tumors with p53 mutation had the lowest values of intra-tumoral CD8^+^ in comparison to the vicinity of the tumor and distant CD8^+^ cells (Figure 4E).

### Analysis of CD3^+^ and CD8^+^ cell densities targeting TCGA subgroups

ROC curve analysis of CD3^+^ and CD8^+^ densities across the three regions of intra-tumoral, close, and distant compared with different TCGA subgroups revealed the best discriminative power for intra-tumoral CD8^+^ densities for the distinction of the POLE status (AUC 0.800, *p* = 0.001). With respect to the opposite data in the NSMP group (AUC 0.325, *p* < 0.001), intra-tumoral CD8^+^ densities show discriminatory power between POLEmut and POLEwt cases.

To achieve a good predictor for POLEwt cases, the optimal cut-off was an intra-tumoral CD8^+^ density as low as 56/mm^2^ in the testing cohort. For pragmatic applicability, a cut-off of 50 truly intra-tumoral CD8^+^ cells per mm^2^ was further on used. Applying this threshold in our model, it excludes 51.2% (129/252) POLE wild-type cases from sequencing with a sensitivity of 100.0% and a specificity of 53.3%. By contrast, it doubles the positive predictive value in secondary sequencing from native 4.0 to 8.1% in a pre-selected series.

### Confirmatory results based on the validation cohort

Of note, data from the validation cohort were based on different TMA core size, staining and scanning protocols. A side-to-side comparison to DAB-stained brightfield immunohistochemistry was not accessible. However, the digital pathology script was exerted identically. Thus, the total cell counts per mm^2^, and the three sub-ordinated regions, were defined identically.

With exception of distant areas, the median cell densities for CD3^+^ and CD8^+^ were each significantly higher than in the testing cohort depicted in Figures 4B, D, F (Mann-Whitney-U-test, each *p* < 0.001 for total areas, intra-tumoral and close). As shown later, this finding affects mainly the specificity of the proposed cut-off.

Regarding the above-mentioned associations of CD3^+^ and CD8^+^ with clinical-pathological parameters, only the TCGA subgroups remained a stable parameter.

The correlation with histological subtype presented with inverse values in comparison to the testing cohort, as endometrioid cases in comparison to non-endometrioid cases showed higher values. This accounts for CD3^+^ with medians of 541/mm^2^ and 115/mm^2^ in comparison to 352/mm^2^ and 7/mm^2^ in close and distant areas, respectively (Mann-Whitney-U-Test *p* = 0.016 and *p* < 0.001). For endometrioid versus non-endometrioid subtype, CD8^+^ revealed median values of 272/mm^2^ versus 140/mm^2^ in total, 358/mm^2^ versus 155/mm^2^ in close subregions, as well as 79/mm^2^ versus 2/mm^2^ in distant regions (Mann-Whitney-U-test, *p* = 0.024, *p* < 0.001, *p* < 0.001, respectively).

In the validation cohort, the analysis of grading revealed opposite data again, as low-grade cases showed significantly higher values of CD3^+^ and CD8^+^ counts than high-grade cases. For CD3^+^ this was shown in comparison between G2 and G3 tumors revealing median differences of 552/mm^2^ to 172/mm^2^ in close regions and 794/mm^2^ to 125/mm^2^ in distant regions (Kruskal-Wallis test *p* = 0.01 and *p* < 0.001 followed by pairwise median test *p* = 0.020 and *p* = 0.010). Regarding CD8^+^, the G1 versus G3 cases showed median values of 355/mm^2^ to 242/mm^2^ in total areas (Kruskal-Wallis test *p* = 0.048 followed by pairwise median test *p* = 0.042), 577/mm^2^ to 290/mm^2^ in close regions (Kruskal-Wallis test *p* < 0.001 followed by pairwise median test *p* < 0.001), and 400/mm^2^ to 43/mm^2^ in distant regions (Kruskal-Wallis test *p* < 0.001 followed by pairwise median test *p* < 0.001), respectively.

Due to the composition of the validation cohort the T-category could only compare between pT1a versus pT1b/pT2 cases, which in intra-tumoral regions showed decreased differences for CD3^+^ with values from 343/mm^2^ to 265/mm^2^ (Mann-Whitney-U test, *p* = 0.031) and CD8^+^ with values from 253/mm^2^ to 155/mm^2^ (Mann-Whitney-U test, *p* = 0.031).

The pN-category could not be tested as the validation cohort consisted of nodal negative cases only. Lymphovascular invasion was only in CD8^+^ close areas associated with significantly decreased medians from 356/mm^2^ in L0 cases to 242/mm^2^ in L1 cases (Mann-Whitney-U test, *p* = 0.048). Hemangioinvasion did not differ in CD3^+^ and CD8^+^ counts. The presence of MELF pattern showed significant decreases from 283/mm^2^ to 176/mm^2^ medians of CD8^+^ in total areas (Mann-Whitney-U-test, *p* = 0.035), which was even more pronounced in close subregions with a decrease from 385/mm^2^ to 247/mm^2^ (Mann-Whitney-U test, *p* = 0.016).

In summary, none of the conventional histopathological parameters was confirmed in its correlation with CD3^+^ and CD8^+^ densities between the testing and validation cohort, indicating that molecular subtype is the main influencing factor on lymphocyte infiltrates.

Regarding TCGA, the validation cohort corroborates the previous findings. As depicted in Figures 4A, B, the distribution patterns appear very similar.

### Cut-off application on the validation cohort

**FIGURE 5:**
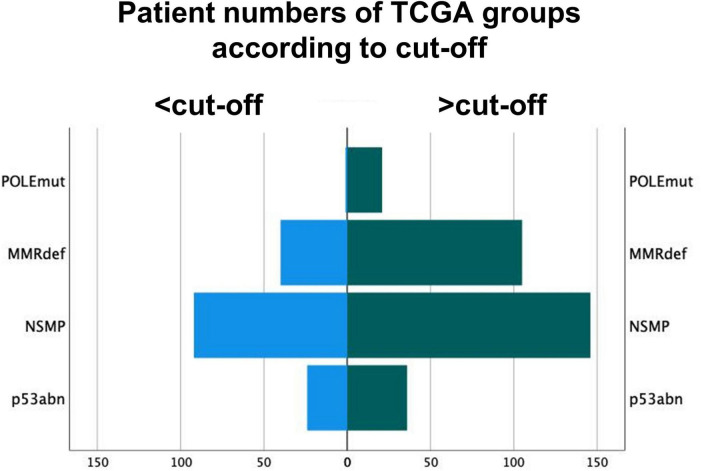
Distribution of molecular subgroups of The Cancer Genome Atlas according to the cut-off of 50 intra-tumoral CD8^+^ lymphocytes per mm^2^. Group 1 are tumors under the cut-off of 50/mm^2^. POLE sequencing might be omitted for these POLE wild-type cases.

Out of the 12 POLE mutated cases in the validation cohort, 11 had an intra-tumoral CD8^+^ mean density of 98/mm^2^ or higher. Hence, the proposed cut-off of 50/mm^2^ CD8^+^ lymphocytes showed a sensitivity of 91.7% in the validation cohort, but a decreased specificity of 13.4% as CD8^+^ counts were generally higher in the validation cohort. Of note, the single POLEmut case as outlier showed exceedingly low values of only 6/mm^2^ in total areas. Combining both cohorts, the cut-off of 50/mm^2^ intra-tumoral CD8^+^ lymphocytes can predict POLEwt cases with a sensitivity of 95.5% and a specificity of 32.3% (Figure 5).

### Prognostic relationship between CD3^+^ and CD8^+^ in comparison to TCGA subgroups

For prognostic analysis both cohorts were combined and tested for conventional histological parameter, TCGA subtypes, medians of CD3^+^ and CD8^+^ densities per region and the above-mentioned cut-off. Information was available for recurrence free survival and overall survival with a follow-up period up to 10 years. As expected, the conventional parameter showed a highly significant stratification of survival data with log-rank test, in detail histological subtype (RFS *p* < 0.001; OS *p* = 0.002), T-category, N-category, grading, lymphovascular and hemangioinvasion (each RFS *p* < 0.001; OS *p* < 0.001). Only MELF pattern did not contribute to prognostics. These findings are consistent with the previous published data (5, 7, 8, 40).

In a first step, CD3^+^ and CD8^+^ counts were analyzed with the median of each subcategory as cut-off for survival analysis. Best stratification was reached for CD3^+^ intra-tumoral (RFS *p* = 0.001; OS *p* < 0.001) and close subregions (RFS *p* = 0.004; OS *p* < 0.001), which also accounted for CD8^+^ in intra-tumoral (RFS *p* = 0.001; OS *p* < 0.001) and close regions (RFS *p* = 0.001; OS *p* < 0.001). Distant regions contributed less to prognostics.

**FIGURE 6:**
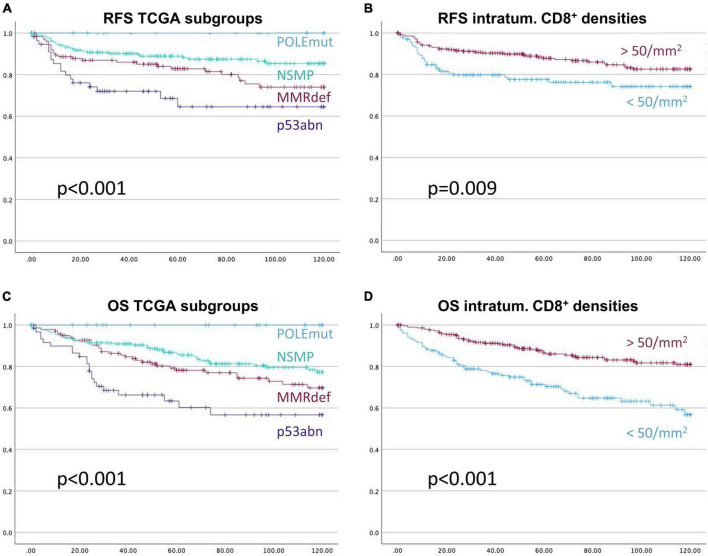
Kaplan-Meier-Curves for recurrence free survival **(A,B)**, and overall survival **(C,D)**, respectively. The prognostic stratification of the subgroups of The Cancer Genome Atlas, **(A,C)**, is more informative than the prognostication with the applied cut-off based on CD8^+^ intra-tumoral cell densities of 50 cells/mm^2^, **(B,D)**. *X*-axis represents month of survival, *y*-axis the proportion of cumulative survival. *P*-values are based on log-rank tests.

Next the above-mentioned cut-off of 50 CD8^+^ cells/mm^2^ was applied to the survival analysis and compared to the performance of TCGA molecular subtypes (Figure 6). Of note, none of the CD3^+^ or CD8^+^ derived parameters exceeded the excellent prognostics POLEmut status can provide.

### Application of the immune infiltrate pretest

**FIGURE 7:**
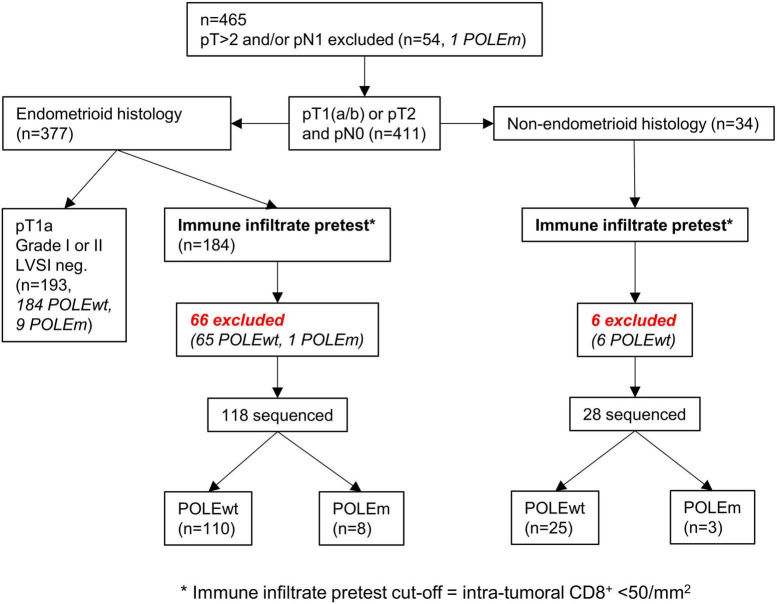
Workflow of applying the cut-off as a pretest to both cohorts. The cut-off of < 50/mm^2^ intra-tumoral CD8^+^ lymphocytes can predict the presence of POLE wild-type tumors.

The cut-off of 50 intra-tumoral CD8^+^ cells per mm^2^ can be integrated and depicted as a pretest within a clinical decision flowchart prior to POLE mutation analysis (Figure 7) (15).

The clinical strategies for reduction of molecular testing further encompass ESGO low-risk cases (e.g., with low grading and no LVSI) and should encounter the existence of multiple classifier carcinomas with combinations of POLE, MMRdef, and p53abn in up to 3% of cases (41).

In total, the immune infiltrate pretest proposes a POLEwt prior to sequencing in 72 (33.0%) from the original 218 cases. If resources and access to molecular testing are limited, the pretest might bring down healthcare costs by a third and nearly doubles the likelihood for positive sequencing results. Together with clinical-pathological assessment, POLE sequencing can be further minimized to only one-third of all cases in our cohorts.

## Discussion

### Diagnostic relevance of the immune infiltrate pretest

The recent WHO classification in EC strengthened the role of molecular characterization in ECs, which represents great progress in the understanding of tumor biological and prognostic differences (1). However, in clinical decision making, preselection of cases to submit for further molecular testing is mandatory in terms of healthcare system cost-effectiveness and timely start of radio- and chemotherapy if necessary. The recently introduced ESGO risk classification shows the value added by molecular data, particularly for FIGO I and II staged ECs (3). First algorithms with immunohistochemistry for MMR proteins and p53, in combination with histological subtype and conventional histopathological parameters, can safely reduce the number of molecular tests by nearly half (3, 15). This is, for instance, because very low-risk cases will not need POLE testing to underline a further decreased risk (15).

POLE mutations are rare, with rates of 6–8% in EC, which urges for even more restrictive pretest developments (2). Tumor-associated immune responses are well described for POLE as well as MMRdef cases (6, 7, 20, 42). Inevitably, the use of immune infiltrate hardly distinguishes MMRdef from POLEmut cases. However, low immune infiltrates remain a predictor of POLE wild-type. Our quantitative and spatial analysis of CD3^+^ and CD8^+^ immune cells as a search tool revealed intra-tumoral CD8^+^ cell densities as the strongest discriminator. For usefulness in daily practice, we consider a cut-off of up to 50/mm^2^ intra-tumoral CD8^+^ lymphocytes as the best applicable value to assume a POLE wild-type situation. Below this threshold, only one POLE mutation was detected. Of note, this patient showed no particularities like immunosuppressive therapies, co-morbidities and had so far no recurrence. In combination with the abovementioned clinical-pathological algorithms, the rate of molecular testing could be further reduced to approximately a third of cases (15).

A recent meta-analysis demonstrated a sensitivity of 85.0% and specificity of 66.0% in detecting POLEmut cases by TILs, if the MMR status was known (43). This is in line with our findings that the TIL reaction grows with increasing tumor mutational burden in terms of hyper-mutational type in MMRdef cases and ultra-mutational type in POLEmut cases (5–8, 44–47). Of note, adding more immune cell markers, as recently performed in extended multi-plexing, might even decipher more discriminators (20).

### Tumor biology of the immune infiltrate

Elevated intra-tumoral cytotoxic CD8^+^ T-cells could indicate a pro-apoptotic reaction that might contribute to the excellent prognosis of POLEmut cases, which is not as pronounced in MMRdef cases (5). Our data imply very basically a biological activation of CD3^+^ cells near a tumor to a recruitment of cytotoxic CD8^+^ cells to the intra-tumoral area, which is most pronounced in POLEmut cases. However, the questions of tumor heterogeneity and other co-founders affecting the immune reaction were not part of this study. Additionally, POLEmut immune induction fits very well to the concept of immune-ablative cancer treatment, but represents a natural course with excellent prognosis even without checkpoint inhibition (47). POLEmut cases with little immune reaction might represent the rare candidates for advanced stages and worse outcome (48). The tumor biological functional roles of TILs in POLEmut cases with respect to checkpoint blockade must be further elucidated. Some studies found higher stromal TILs than intraepithelial, and one study found higher TILs in MMRdef tumors compared to POLE (7, 8, 49), which can be explained by the similarities of ultra- and hyper-mutated cancers with possibly similar tumor mutational burden.

### Analysis with conventional parameters and prognosis

Relating to conventional prognosticators, no consistencies were found in comparison between histological subtype, grading, and TNM-classification, including LVI status. In tendency, a positive correlation between grading and TILs has been described but might be influenced by the existence of serous or clear cell carcinomas (40, 49–51).

The prognostic effect of TILs has been widely discussed as pro (49, 51, 52), and contra (7, 8, 40). Since there is a positive correlation between POLE mutated tumors and a higher CD8^+^ intra-tumoral immune infiltrate (5–8, 14, 44–46), it would be logical that their prognostic significance might be dependent on case numbers of POLE mutated ECs. This has recently been shown by a meta-analysis of almost two thousand EC cases (53). Additionally, our data elucidate a stronger role of CD3^+^ and CD8^+^ cell densities in intra-tumoral and close subregions on prognosis. Hence, more precise definitions and distinctions of intra-tumoral tumor infiltrating lymphocytes (iTILs) from stromal tumor infiltrating lymphocytes (sTILs) are warranted.

### Limitations and strengths

POLE sequencing remains the gold standard for appropriate molecular subtyping as outliers based on immune cell densities like in our study occur (47). Together with the validation cohort, we found less than five per cent POLEmut cases, which represents a low rate of POLEmut according to literature (6–8, 39, 49). If real-life rates of POLEmut remain as low, increased pretest probabilities might justify POLE sequencing costs and expand its use in healthcare systems with more limited resources. Additionally, risk factors justifying molecular subtyping might only be evident after surgery and timely indication for radio- or chemotherapy might be compromised, if molecular analysis is delayed. In such situations conventional pretests might point toward therapeutic options until sequencing information is available.

Another limitation is the complexity of multiplexed immunofluorescence, which is not yet used as a standard staining technique for daily diagnostic usage.

The differences between both cohorts in cell counts and results for histological subtype and grading could only partially be explained by the clinical-pathological differences, and are also attributable to the different staining protocols and scanning conditions. This is reflected in the available literature as well. Different studies show great ranges in intra-tumoral and stromal CD8^+^ densities between 14/mm^2^ and 650/mm^2^ (Supplementary material) (5, 6, 8, 9, 52, 54). To properly apply the immune infiltrate pretest, it would be necessary to harmonize staining conditions.

A strength is the high correlation of the testing cohort immune infiltrate counts between the fluorescence method and the gold standard; conventional DAB staining validated for the Immunoscore. As a consequence, we maintained the cut-off from the testing cohort as a reference point. However, the variability in immunohistochemistry between laboratories might need round-robin tests or even laboratory specific reference values from other biomarkers (20). Additionally, our proposed cut-off needs validation in clinical trials before clinical usage.

## Conclusion

Our spatial multiplex immunofluorescence approach could decipher a possible diagnostic test for clinical decision making in EC. An intra-tumoral CD8^+^ lymphocyte density of less than 50/mm^2^ predicts very likely a POLE wild-type situation, so this cut-off might be used as a pretest to avoid expensive molecular subtyping. Our method shows the potential to be transferred to brightfield applications in daily routine, to improve clinical decision making, and to reduce healthcare costs.

## Data availability statement

The raw data supporting the conclusions of this article will be made available by the authors, without undue reservation.

## Ethics statement

The studies involving human participants were reviewed and approved by Ethics Committee Bern, Switzerland (reference number: 2018-00479) and Ethics Committee University Hospital La Paz (reference number: HULP#PI3778). The patients/participants provided their written informed consent to participate in this study.

## Author contributions

TR developed the study concept and design, performed the histopathological data-monitoring and case evaluation together with LC, originally created the ngTMA^®^ slides, supervised the process, and was responsible for co-writing and editing. SI, FS, and MMü were responsible for patient recruiting and gathering of clinical data. JC performed the POLE hotspot sequencing and classification of VUS. JG and SJ stained and scanned the slides for multiplex immunofluorescence. LN and SJ wrote the script for digital image analysis. SJ acquired and set up the raw data and prepared the figures and tables, and primarily wrote the manuscript. TR and IZ made the statistics. TP, VH-S, IR-C, DH, AR, and MMe provided resources of the validation cohort and helped with conceptualization and methodology of including data of the validation cohort into the manuscript. All authors contributed to the article and approved the submitted version.

## Supplementary material

Supplementary Figure 1 Description of the script workflow for digital image analysis. **(A)** Tissue detection. **(B)** Cell detection. **(C)** Tumor detection. **(D)** Defining three different compartments intra-tumoral, tumor neighborhood (< 50 microns away from tumor), and tumor distant (> 50 microns away from tumor). **(E)** Allocating cells to classes in every compartment. Stromal cells (blue), CD3^+^ cells (green), CD8^+^ cells (red), and tumor cells (brown).

Supplementary Figure 2 Comparison of multiplexed immunofluorescence **(A,C,E)** and digital image analysis **(B,D,F)** in the testing cohort. Note the CD3^+^ cells (green) and CD8^+^ cells (red).

Supplementary Figure 3 Comparison of multiplexed immunofluorescence **(A,C,E)** and digital image analysis **(B,D,F)** in the validation cohort. Due to different staining and scanning conditions, CD8^+^ cells appear green.

Supplementary Table 1 Literature search of available studies with CD8^+^ cell densities. If possible, density values were converted to mm^2^ for better comparability. Note that CD8^+^ cell densities vary between 14/mm^2^ and 650/mm^2^.

Supplementary Data Sheet 1 Script checklist and full scripts used for digital image analysis. The scripts were written in Groovy programming language suitable for the QuPath software. They allow an automated analysis of lymphocyte cell counts, cell percentages, and cell densities in three compartments of a TMA core.
