# Supplementary figures and images for: Spatial distribution of CD3- and CD8-positive lymphocytes as pretest for POLE wild-type in molecular subgroups of endometrial carcinoma
Source: Front Med (Lausanne). 2023 Mar 23;10:1110529. doi: 10.3389/fmed.2023.1110529 (PMC10076655; doi:10.3389/fmed.2023.1110529)

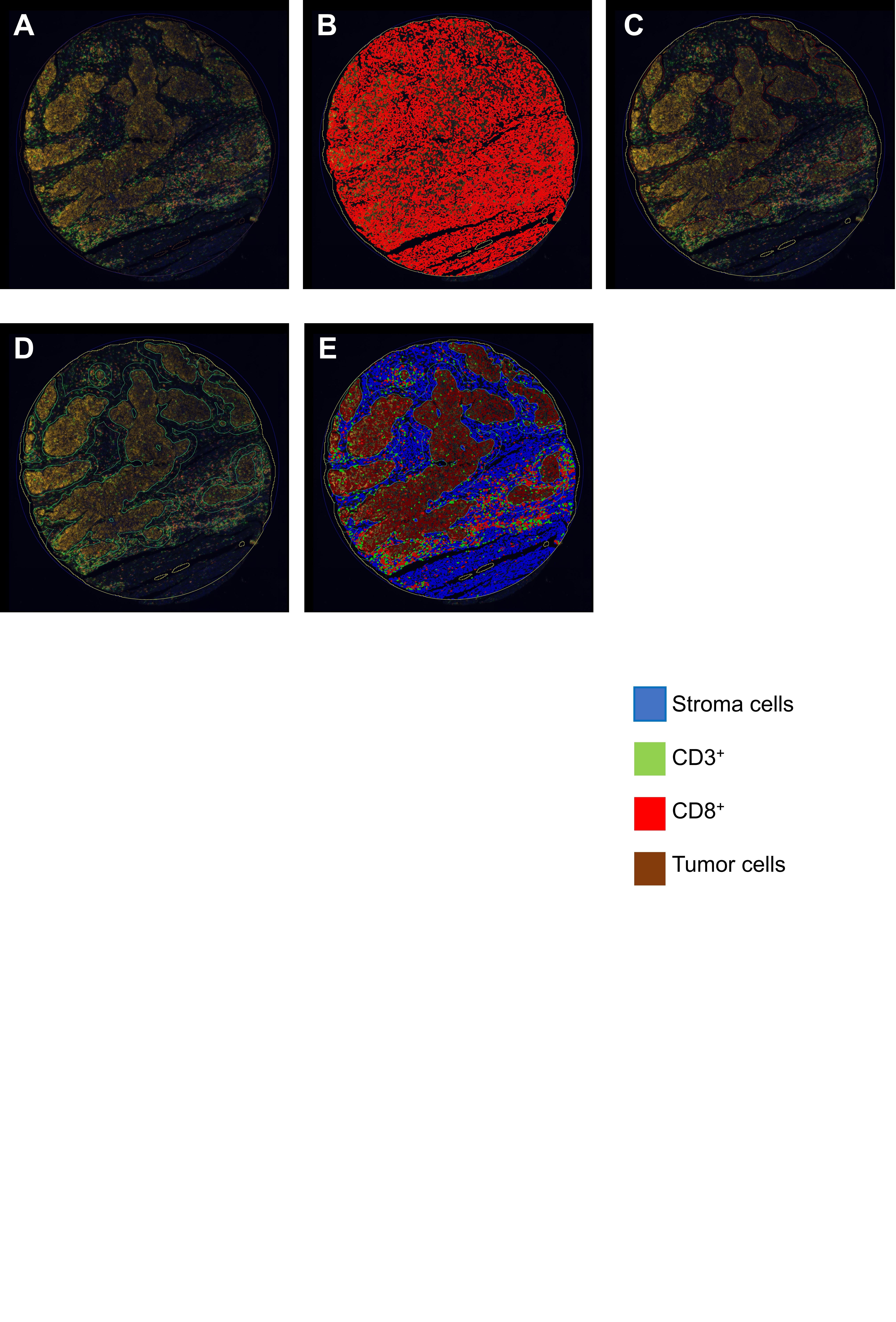

Supplement: Supplementary Figure 1 — Description of the script workflow for digital image analysis. (A) Tissue detection. (B) Cell detection. (C) Tumor detection. (D) Defining three different compartments intra-tumoral, tumor neighborhood (< 50 microns away from tumor), and tumor distant (> 50 microns away from tumor). (E) Allocating cells to classes in every compartment. Stromal cells (blue), CD3+ cells (green), CD8+ cells (red), and tumor cells (brown). [file Image_1.JPEG]

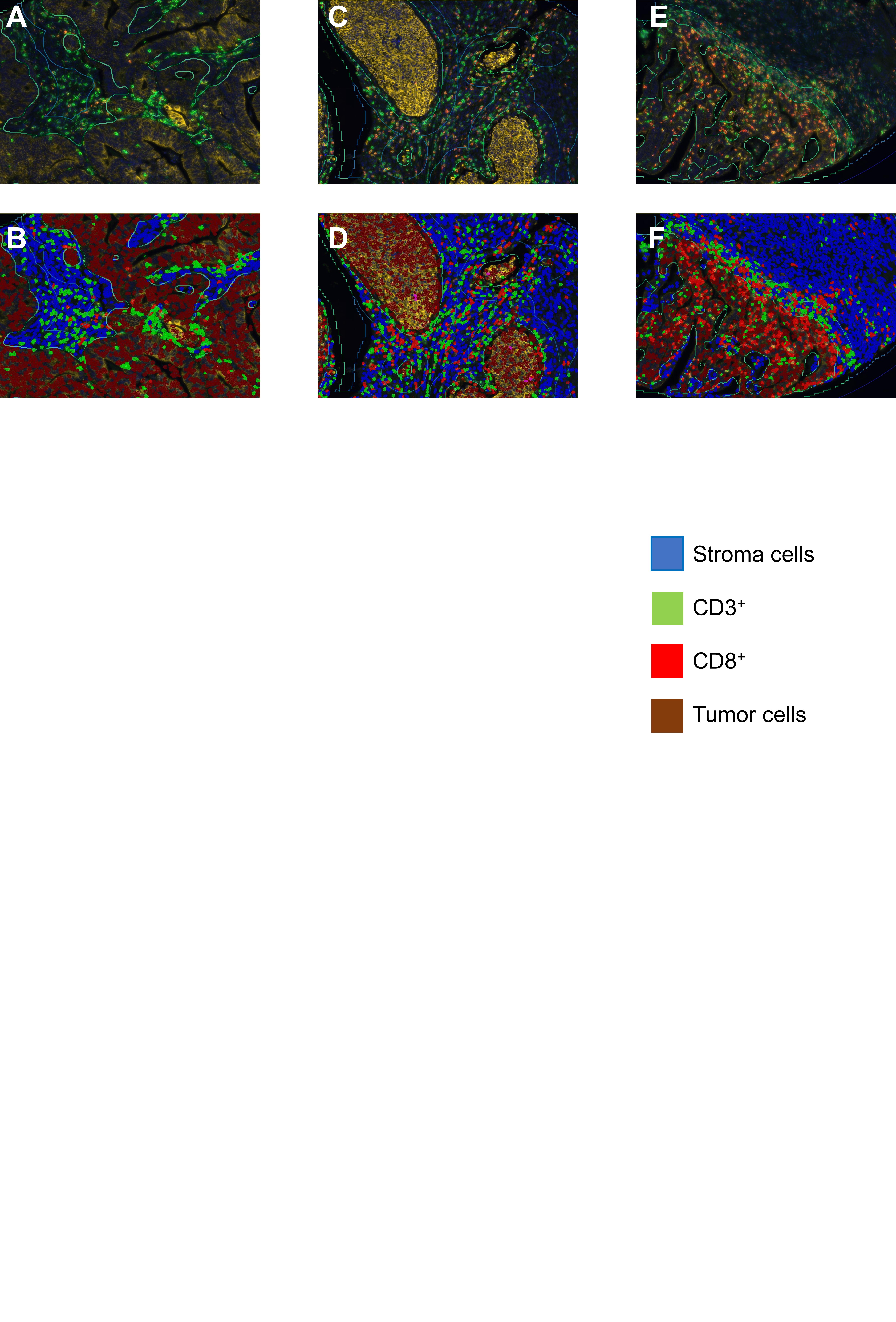

Supplement: Supplementary Figure 2 — Comparison of multiplexed immunofluorescence (A,C,E) and digital image analysis (B,D,F) in the testing cohort. Note the CD3+ cells (green) and CD8+ cells (red). [file Image_2.JPEG]

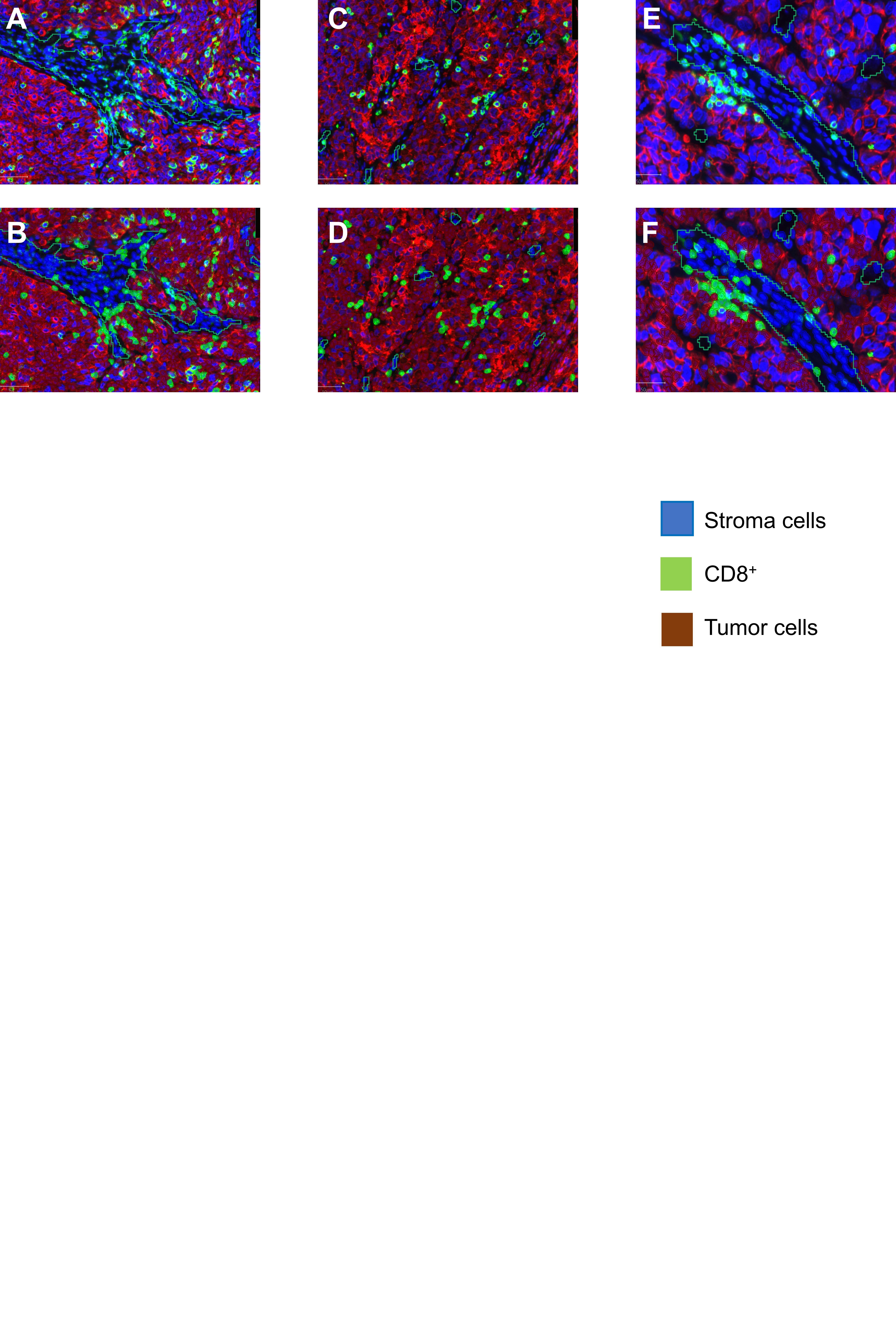

Supplement: Supplementary Figure 3 — Comparison of multiplexed immunofluorescence (A,C,E) and digital image analysis (B,D,F) in the validation cohort. Due to different staining and scanning conditions, CD8+ cells appear green. [file Image_3.JPEG]
