# Supplementary material for: Spatial distribution of CD3- and CD8-positive lymphocytes as pretest for POLE wild-type in molecular subgroups of endometrial carcinoma
Source: Front Med (Lausanne). 2023 Mar 23;10:1110529. doi: 10.3389/fmed.2023.1110529 (PMC10076655; doi:10.3389/fmed.2023.1110529)
Supplement: Supplementary Table 1 — Literature search of available studies with CD8+ cell densities. If possible, density values were converted to mm2 for better comparability. Note that CD8+ cell densities vary between 14/mm2 and 650/mm2. [file Table_1.pdf]

| Study | PubMed ID | CD8⁺ cell values | Converted to mm² |
|---|---|---|---|
| De Jong 2009 | 19411095 | Cut-off = 4/0.283mm²<br><br><4 = low, >4 = high | **14.3/mm²** |
| Cermakova 2014 | 25275055 | intraepithelial:<br>15+-17/mm²<br><br>stromal:<br>47+-42/mm² | <br>**15+-17/mm²**<br><br><br>**47+-42/mm²** |
| Howitt 2015 | 26181000 | POLE and MSI 32.8/HPF<br><br>MSS 13.5/HPF<br><br>HPF defined as 40x objective, no mm² | N.C. |
| Van Gool 2015 | 25878334 | intraepithelial:<br>POLE 5.9/HPF<br>MSI 2.6/HPF<br><br>stromal:<br>POLE 26/HPF<br>MSI 13.5/HPF<br><br>HPF defined as 200x200 um | <br>POLE **147.5/mm²**<br>MSI **65/mm²**<br><br><br>POLE **650/mm²**<br>MSI **325/mm²** |
| Mehnert 2016 | 27159395 | Score 1-3 with lymphocytic invasion<br><br>POLE with highest Infiltration, no numbers | N.C. |
| Eggink 2017 | 28344870 | 80 cells/core<br><br>Cores with 1.0 mm diameter | **101.6/mm²** |
| Crumley 2019 | 30291344 | 398/mm² PD-L1 negative<br><br>571/mm² PD-L1 positive | **398/mm²** PD-L1 negative<br><br>**571/mm²** PD-L1 positive |
| Talhouk 2019 | 30523022 | intraepithelial:<br>POLE 4.8 (27.9)<br>MMRdef 4.2 (18.4)<br>NSMP 2.6 (6)<br>p53abn 2.7 (6.5)<br><br>stromal:<br>POLE 5.8 (55.7)<br>MMRdef 5.2 (36.8)<br>NSMP 3.9 (14.9)<br>p53abn 3.9 (14.9)<br><br>log(2) based, cores with 0.6 mm diameter | total :<br>POLE **298.5/mm²**<br>MMRdef **197/mm²**<br>NSMP **74.6/mm²**<br>p53abn **76.4/mm²** |
| Horeweg 2020 | 32999003 | intraepithelial:<br>31.1 cells/mm²<br><br>stromal:<br>41.9 cells/mm² | <br>**31.1/mm²**<br><br><br>**41.9/mm²** |
| Willvonseder 2021 | 33340331 | intraepithelial:<br>7.45%<br><br>stromal:<br>16.22%<br><br>Mean cell densities/100 cells | N.C. |
