# Supplementary material for: Spatial distribution of CD3- and CD8-positive lymphocytes as pretest for POLE wild-type in molecular subgroups of endometrial carcinoma
Source: Front Med (Lausanne). 2023 Mar 23;10:1110529. doi: 10.3389/fmed.2023.1110529 (PMC10076655; doi:10.3389/fmed.2023.1110529)
Supplement: Supplementary Data Sheet 1 — Script checklist and full scripts used for digital image analysis. The scripts were written in Groovy programming language suitable for the QuPath software. They allow an automated analysis of lymphocyte cell counts, cell percentages, and cell densities in three compartments of a TMA core. [file Data_Sheet_1.docx]

**Script checklist**

1. Remove items like annotations and metadata
2. Create tissue annotation
3. Detect cells in tissue annotation
4. Create measurements for CD3, CD8 and CK (testing cohort only)
5. Create tumor annotation
6. Split tissue and tumor annotation into three compartments: intra-tumoral, close and distant
7. Set path classes for tumor, stroma, CD3^+^ and CD8^+^ cells
8. Create measurements like number, percentage and density for every cell type in the three compartments of a core
9. Export measurements into an excel file

**Full script used for digital image analysis of testing cohort**

/**

* Apply on image with markers to be investigated.

*@authors Luca Noti, Samuel Jungen

*/

// Import, definitions

import static qupath.lib.gui.scripting.QPEx.*

import qupath.lib.objects.PathAnnotationObject;

import qupath.lib.analysis.DistanceTools

import qupath.lib.common.GeneralTools

import qupath.lib.objects.PathObject

import qupath.lib.objects.PathObjects

import qupath.lib.objects.PathCellObject

import qupath.lib.roi.GeometryTools

import qupath.lib.roi.ROIs

import org.locationtech.jts.geom.Geometry

def imageData = getCurrentImageData()

def hierarchy = imageData.getHierarchy()

def server = imageData.getServer()

def tissue = getPathClass("Tissue")

def tumor = getPathClass("Tumor")

def stromaTN = getPathClass("Stroma TN")

def stromaTD = getPathClass("Stroma TD")

def stromaNeg = getPathClass("Stroma Negative")

def stromaCD3 = getPathClass("Stroma CD3")

def stromaCD8 = getPathClass("Stroma CD8")

// Pixel size

def pixelSize = server.getPixelCalibration().getAveragedPixelSizeMicrons()

// Image settings

setImageType('FLUORESCENCE')

// Print Image name

def name = getProjectEntry().getImageName()

print("Running script for slide " + name)

// Remove items

clearAnnotations()

resetTMAMetadata(true)

print("Items removed!")

// Check if TMA is dearrayed

if (!isTMADearrayed()) {

print ("Please dearray TMA first!")

return;

}

// Create tissue annotations from thresholder

selectTMACores()

createAnnotationsFromPixelClassifier("Tissue_Thresholder", 1000.0, 0.0)

print ("Tissue annotations created!")

// Shrink Tissue annotations by 1 pixel for correct hierarchy

tissueShrinked = []

for (a in getAnnotationObjects().findAll{it.getPathClass() == getPathClass("Tissue")}) {

aClass = a.getPathClass()

// Creating ROI

def roi = a.getROI()

// Creating Geometrie (= Area)

def geom = roi.getGeometry()

def plane = roi.getImagePlane()

// Shrinking Annotation

geom = geom.buffer(-1)

geom = GeometryTools.homogenizeGeometryCollection(geom)

roi = GeometryTools.geometryToROI(geom, plane)

anew = PathObjects.createAnnotationObject(roi)

// Adding new Annotation

anew.setPathClass(aClass)

anew.setLocked(true)

tissueShrinked.add(anew)

}

removeObjects(getAnnotationObjects().findAll{it.getPathClass() == getPathClass("Tissue")}, true)

hierarchy.insertPathObjects(tissueShrinked)

print("Shrinked Tissue annotations created")

// Detect cells (mean thresholding)

selectAnnotations().findAll{it.getPathClass() == tissue}

runPlugin('qupath.imagej.detect.cells.WatershedCellDetection', '{"detectionImage": "Channel 3", "requestedPixelSizeMicrons": 0.65, "backgroundRadiusMicrons": 0.0, "medianRadiusMicrons": 0.0, "sigmaMicrons": 1.5, "minAreaMicrons": 10.0, "maxAreaMicrons": 50.0, "threshold": 35.0, "watershedPostProcess": true, "cellExpansionMicrons": 2.0, "includeNuclei": true, "smoothBoundaries": true, "makeMeasurements": true}');

print("Cells detected!")

// Create CD3 measurement

String m1 = "Cytoplasm: Channel 2 max" //CD3 Peak (exclude autofluorescence from erythrocytes)

String m2 = "Cytoplasm: Channel 2 mean" //CD3 constant signal

String m3 = "Nucleus: Channel 3 mean" //DAPI Background

def th1 = 96

def th2 = 36

def th3 = 35

for (cell in getDetectionObjects()) {

def ml = cell.getMeasurementList()

double cellm1 = measurement(cell, m1)

double cellm2 = measurement(cell, m2)

double cellm3 = measurement(cell, m3)

def pos = -1

if (cellm1 >= th1 && cellm2 >= th2 && cellm3 >= th3) {

pos = 1

}

else {

pos = 0

}

ml.putMeasurement("CD3", pos)

ml.close()

}

print("CD3 measurement created!")

// Create CD8 measurement

String m4 = "Cytoplasm: Channel 1 max" //CD8 Peak (exclude autofluorescence from erythrocytes)

String m5 = "Cytoplasm: Channel 1 mean" //CD8 constant signal

String m6 = "Nucleus: Channel 3 mean" //DAPI Background

def th4 = 116

def th5 = 33

def th6 = 35

for (cell in getDetectionObjects()) {

def ml = cell.getMeasurementList()

double cellm4 = measurement(cell, m4)

double cellm5 = measurement(cell, m5)

double cellm6 = measurement(cell, m6)

def pos = -1

if (cellm4 >= th4 && cellm5 >= th5 && cellm6 >= th6) {

pos = 1

}

else {

pos = 0

}

ml.putMeasurement("CD8", pos)

ml.close()

}

print("CD8 measurement created!")

// Create CK measurement

String m7 = "Cytoplasm: Channel 4 mean" //CK Background (exclude autofluorescence from tumor)

def th7 = 61

for (cell in getDetectionObjects()) {

def ml = cell.getMeasurementList()

double cellm7 = measurement(cell, m7)

def pos = -1

if (cellm7 >= th7) {

pos = 1

}

else {

pos = 0

}

ml.putMeasurement("CK", pos)

ml.close()

}

print("CK measurement created!")

// Create tumor annotations from thresholder

selectAnnotations().findAll{it.getPathClass() == getPathClass("Tissue")}

createAnnotationsFromPixelClassifier("Tumor_Thresholder", 1000.0, 0.0)

print ("Tumor annotations created!")

// Shrink Tumor annotations by 1 pixel for correct hierarchy

tumorShrinked = []

for (a in getAnnotationObjects().findAll{it.getPathClass() == getPathClass("Tumor")}) {

aClass = a.getPathClass()

// Creating ROI

def roi = a.getROI()

// Creating Geometrie (= Area)

def geom = roi.getGeometry()

def plane = roi.getImagePlane()

// Shrinking Annotation

geom = geom.buffer(-1)

geom = GeometryTools.homogenizeGeometryCollection(geom)

roi = GeometryTools.geometryToROI(geom, plane)

anew = PathObjects.createAnnotationObject(roi)

// Adding new Annotation

anew.setPathClass(aClass)

anew.setLocked(true)

tumorShrinked.add(anew)

}

removeObjects(getAnnotationObjects().findAll{it.getPathClass() == getPathClass("Tumor")}, true)

hierarchy.insertPathObjects(tumorShrinked)

print("Shrinked Tumor annotations created")

// Create Stroma TN and Stroma TD annotations

//**

def radiusMicronsTN = 50.0

double expandPixelsTN = radiusMicronsTN / pixelSize

def cores = hierarchy.getTMAGrid().getTMACoreList()

for (c in cores){

if (cores.indexOf(c)% 10 == 0){

print("Processing Tumor Neighborhood annotations for core nr. " + (cores.indexOf(c)+1))

}

def annotationsToAdd = []

// Instanciate PathObjects, ROIs, Geometries

PathObject annotationTissue

PathObject annotationTumor

PathObject annotationTN

PathObject annotationTD

//ROI roiStroma

//ROI roiTumor

//ROI roiTN

//ROI roiTD

Geometry geomTissue

Geometry geomTumor

Geometry geomTN

Geometry geomTD

def plane

// Cores with NO tissue annotation

if (c.getDescendantObjects().findAll{it.getPathClass() == tissue && it.isAnnotation() == true} == []){

print("Core without Tissue found: " + c)

continue

}

// Cores with tissue AND tumor annotations

else if ((c.getDescendantObjects().findAll{it.getPathClass() == tumor && it.isAnnotation() == true} != []) && (c.getDescendantObjects().findAll{it.getPathClass() == tissue && it.isAnnotation() == true} != [])) {

// Create Stroma TN annotation

// Creating PathObjects

annotationTissue = c.getDescendantObjects().findAll{it.getPathClass() == tissue && it.isAnnotation() == true}.get(0)

annotationTumor = c.getDescendantObjects().findAll{it.getPathClass() == tumor && it.isAnnotation() == true}.get(0)

// Creating ROIs

def roiTissue = annotationTissue.getROI()

def roiTumor = annotationTumor.getROI()

// Creating Geometries

geomTissue = roiTissue.getGeometry()

geomTumor = roiTumor.getGeometry()

plane = roiTumor.getImagePlane()

// Creating new Geometries and Stroma TN Annotations

geomTN = geomTumor.buffer(expandPixelsTN)

geomTN = geomTN.difference(geomTumor)

geomTN = geomTN.intersection(geomTissue)

geomTN = GeometryTools.homogenizeGeometryCollection(geomTN)

def roiTN = GeometryTools.geometryToROI(geomTN, plane)

annotationTN = PathObjects.createAnnotationObject(roiTN)

annotationTN.setPathClass(stromaTN)

annotationTN.setLocked(true)

if (roiTN.getArea() > 0){

annotationsToAdd.add(annotationTN)

}

// Creating new Geometries and Stroma TD Annotations

geomTD = geomTissue.difference(geomTumor)

geomTD = geomTD.difference(geomTN)

geomTD = GeometryTools.homogenizeGeometryCollection(geomTD)

def roiTD = GeometryTools.geometryToROI(geomTD, plane)

annotationTD = PathObjects.createAnnotationObject(roiTD)

annotationTD.setPathClass(stromaTD)

annotationTD.setLocked(true)

if (roiTD.getArea() > 0){

annotationsToAdd.add(annotationTD)

}

// Deleteing Tissue annotation

removeObject(annotationTissue, true)

print("TN and TD created")

}

// Cores with ONLY Tissue annotations (without tumor)

else if ((c.getDescendantObjects().findAll{it.getPathClass() == tumor && it.isAnnotation() == true} == []) && (c.getDescendantObjects().findAll{it.getPathClass() == tissue && it.isAnnotation() == true} != [])) {

annotationTissue = c.getDescendantObjects().findAll{it.getPathClass() == tissue && it.isAnnotation() == true}.get(0)

annotationTD = annotationTissue

annotationTD.setPathClass(stromaTD)

annotationTD.setLocked(true)

annotationsToAdd.add(annotationTD)

// Deleteing Tissue annotation

removeObject(annotationTissue, true)

print("Only TD created")

}

// Adding 3 Annotations into core hierarchy(Stroma TN, Stroma TD, Stroma TI)

// Using this method will sort hierarchy already! No need for resolveHierarchy() afterwards

if (annotationsToAdd != null){

hierarchy.insertPathObjects(annotationsToAdd)

print(c)

print(annotationsToAdd)

}

}

print("Spatial annotations created for " + cores.size() + " cores!")

// Removing parentless Annotations (= Polylines without area +- Tumor?)

def annotationsToRemove = getAnnotationObjects().findAll{it.getLevel() == 1}

print("Removing parentles annotations: " + annotationsToRemove)

removeObjects(annotationsToRemove, true)

// Allocate cells according to PathClass

for (cell in getDetectionObjects()) {

def CD3 = measurement(cell, "CD3")

def CD8 = measurement(cell, "CD8")

def CK = measurement(cell, "CK")

// Classify tumor cells

if ((cell.getParent().getPathClass() == tumor) && (CD3 == 0) && (CD8 == 0)) {

cell.setPathClass(tumor)

}

// Classify negative stroma cells

else if ((CD3 == 0) && (CD8 == 0)) {

cell.setPathClass(stromaNeg)

}

// Classify CD3 stroma cells

else if ((CK == 0) && (CD3 == 1) && (CD8 == 0)) {

cell.setPathClass(stromaCD3)

}

// Classify CD8 stroma cells

else if ((CK == 0) && (CD8 == 1)) {

cell.setPathClass(stromaCD8)

}

}

// Create measurements for annotations

Set classList = []

for (object in getAllObjects().findAll{it.isDetection()}) {

classList << object.getPathClass()

}

for (annotation in getAnnotationObjects()){

def totalCells = []

def ml = annotation.getMeasurementList()

double annotationArea = annotation.getROI().getArea() * pixelSize * pixelSize / 1000000

totalCells = annotation.getDescendantObjects().findAll{it.isDetection() == true}

ml.putMeasurement("Area mm^2", annotationArea)

ml.putMeasurement("Total cells", totalCells.size())

for (aClass in classList){

if (aClass){

if (totalCells.size() > 0){

def cells = totalCells.findAll{it.getPathClass() == aClass}

ml.putMeasurement(aClass.getName()+" cells Num", cells.size())

ml.putMeasurement(aClass.getName()+" cells %", cells.size()*100/totalCells.size())

ml.putMeasurement(aClass.getName()+" cells /mm^2", cells.size() / annotationArea)

ml.close()

}

else {

ml.putMeasurement(aClass.getName()+" Num cells", 0)

ml.putMeasurement(aClass.getName()+" %", 0)

ml.putMeasurement(aClass.getName()+" cells /mm^2", 0)

ml.close()

}

}

}

}

print("Annotation measurements calculated!")

// Edit TMA measurement list

for (c in cores){

def ml = c.getMeasurementList()

for (annotation in c.getChildObjects().findAll{it.isAnnotation() == true}){

ml.putMeasurement(annotation.getPathClass().toString() + ": Total cells", measurement(annotation, "Total cells"))

ml.putMeasurement(annotation.getPathClass().toString() + ": Area mm^2", measurement(annotation, "Area mm^2"))

for (aClass in classList){

String classNum = aClass.getName().toString() + " cells Num"

ml.putMeasurement(annotation.getPathClass().toString() + ": " + aClass.getName() + " cells Num", measurement(annotation, classNum))

String classPercent = aClass.getName().toString() + " cells %"

ml.putMeasurement(annotation.getPathClass().toString() + ": " + aClass.getName() + " cells %", measurement(annotation, classPercent))

String classDensity = aClass.getName().toString() + " cells /mm^2"

ml.putMeasurement(annotation.getPathClass().toString() + ": " + aClass.getName() + " cells /mm^2", measurement(annotation, classDensity))

}

}

ml.close()

}

print("TMA measurements created!")

// Export TMA Measurements

saveTMAMeasurements("D:/Testing cohort/Results")

print("TMA measurements exported!")

**Full script used for digital image analysis of validation cohort**

/**

* Apply on image with markers to be investigated

* @authors Luca Noti, Samuel Jungen

*/

// Import, definitions

import static qupath.lib.gui.scripting.QPEx.*

import qupath.lib.objects.PathAnnotationObject;

import qupath.lib.analysis.DistanceTools

import qupath.lib.common.GeneralTools

import qupath.lib.objects.TMACoreObject

import qupath.lib.roi.interfaces.ROI

import qupath.lib.objects.hierarchy.DefaultTMAGrid

import qupath.lib.roi.RectangleROI

import qupath.lib.objects.PathObject

import qupath.lib.objects.PathObjects

import qupath.lib.objects.PathCellObject

import qupath.lib.roi.GeometryTools

import qupath.lib.roi.ROIs

import org.locationtech.jts.geom.Geometry

def imageData = getCurrentImageData()

def hierarchy = imageData.getHierarchy()

def server = imageData.getServer()

def tissue = getPathClass("Tissue")

def tumor = getPathClass("Tumor")

def stromaTN = getPathClass("Stroma TN")

def stromaTD = getPathClass("Stroma TD")

def stromaNeg = getPathClass("Stroma Negative")

def stromaCD3 = getPathClass("Stroma CD3")

def stromaCD8 = getPathClass("Stroma CD8")

// Pixel size

setPixelSizeMicrons(0.34, 0.34)

def pixelSize = server.getPixelCalibration().getAveragedPixelSizeMicrons()

// Image settings

setImageType('FLUORESCENCE')

// User Input (ImageTypes: "ellipse", "rectangle", null (= none of the above, e.g. full TMA))

String imageType = "ellipse";

// Print Image name

def name = getProjectEntry().getImageName()

print("Running script for slide " + name)

// TMA Preparations

if (imageType == "ellipse" || imageType == "rectangle"){

int widthPixels = server.getWidth()

int heightPixels = server.getHeight()

int diameterNewCore

PathObject c = new TMACoreObject()

if (imageType == "ellipse"){

diameterNewCore = Math.max(widthPixels, heightPixels)

c = PathObjects.createTMACoreObject((widthPixels / 2), (heightPixels / 2), diameterNewCore, false)

}

if (imageType == "rectangle"){

ROI roi = new RectangleROI(0, 0, widthPixels, heightPixels)

c = new TMACoreObject(roi, false)

}

def tmaGrid = new DefaultTMAGrid([c], 1)

hierarchy.setTMAGrid(tmaGrid)

}

// Remove items

clearAnnotations()

resetTMAMetadata(true)

print("Items removed!")

// Check if TMA is dearrayed

if (!isTMADearrayed()) {

print ("Please dearray TMA first!")

return;

}

// Create tissue annotations from thresholder

selectTMACores()

createAnnotationsFromPixelClassifier("Tissue_Thresholder", 1000.0, 0.0)

print ("Tissue annotations created!")

// Shrink Tissue annotations by 1 pixel for correct hierarchy

tissueShrinked = []

for (a in getAnnotationObjects().findAll{it.getPathClass() == getPathClass("Tissue")}) {

aClass = a.getPathClass()

// Creating ROI

def roi = a.getROI()

// Creating Geometrie (= Area)

def geom = roi.getGeometry()

def plane = roi.getImagePlane()

// Shrinking Annotation

geom = geom.buffer(-1)

geom = GeometryTools.homogenizeGeometryCollection(geom)

roi = GeometryTools.geometryToROI(geom, plane)

anew = PathObjects.createAnnotationObject(roi)

// Adding new Annotation

anew.setPathClass(aClass)

anew.setLocked(true)

tissueShrinked.add(anew)

}

removeObjects(getAnnotationObjects().findAll{it.getPathClass() == getPathClass("Tissue")}, true)

hierarchy.insertPathObjects(tissueShrinked)

print("Shrinked Tissue annotations created")

// Detect cells (mean thresholding)

selectAnnotations().findAll{it.getPathClass() == tissue}

runPlugin('qupath.imagej.detect.cells.WatershedCellDetection', '{"detectionImage": "Channel 3", "requestedPixelSizeMicrons": 0.34, "backgroundRadiusMicrons": 0.0, "medianRadiusMicrons": 0.0, "sigmaMicrons": 1.5, "minAreaMicrons": 10.0, "maxAreaMicrons": 50.0, "threshold": 2364, "watershedPostProcess": true, "cellExpansionMicrons": 2.0, "includeNuclei": true, "smoothBoundaries": true, "makeMeasurements": true}');

print("Cells detected!")

// Create CD3 measurement

String m1 = "Cytoplasm: Channel 4 max" //CD3 Peak (exclude autofluorescence from erythrocytes)

String m2 = "Cytoplasm: Channel 4 mean" //CD3 constant signal

String m3 = "Nucleus: Channel 3 mean" //DAPI Background

def th1 = 5595

def th2 = 1968

def th3 = 2364

for (cell in getDetectionObjects()) {

def ml = cell.getMeasurementList()

double cellm1 = measurement(cell, m1)

double cellm2 = measurement(cell, m2)

double cellm3 = measurement(cell, m3)

def pos = -1

if (cellm1 >= th1 && cellm2 >= th2 && cellm3 >= th3) {

pos = 1

}

else {

pos = 0

}

ml.putMeasurement("CD3", pos)

ml.close()

}

print("CD3 measurement created!")

// Create CD8 measurement

String m4 = "Cytoplasm: Channel 2 max" //CD8 Peak (exclude autofluorescence from erythrocytes)

String m5 = "Cytoplasm: Channel 2 mean" //CD8 constant signal

String m6 = "Nucleus: Channel 3 mean" //DAPI Background

def th4 = 9161

def th5 = 1213

def th6 = 2364

for (cell in getDetectionObjects()) {

def ml = cell.getMeasurementList()

double cellm4 = measurement(cell, m4)

double cellm5 = measurement(cell, m5)

double cellm6 = measurement(cell, m6)

def pos = -1

if (cellm4 >= th4 && cellm5 >= th5 && cellm6 >= th6) {

pos = 1

}

else {

pos = 0

}

ml.putMeasurement("CD8", pos)

ml.close()

}

print("CD8 measurement created!")

// Create tumor annotations from thresholder

selectAnnotations().findAll{it.getPathClass() == getPathClass("Tissue")}

createAnnotationsFromPixelClassifier("Tumor_Thresholder", 1000.0, 0.0)

print ("Tumor annotations created!")

// Shrink Tumor annotations by 1 pixel for correct hierarchy

tumorShrinked = []

for (a in getAnnotationObjects().findAll{it.getPathClass() == getPathClass("Tumor")}) {

aClass = a.getPathClass()

// Creating ROI

def roi = a.getROI()

// Creating Geometrie (= Area)

def geom = roi.getGeometry()

def plane = roi.getImagePlane()

// Shrinking Annotation

geom = geom.buffer(-1)

geom = GeometryTools.homogenizeGeometryCollection(geom)

roi = GeometryTools.geometryToROI(geom, plane)

anew = PathObjects.createAnnotationObject(roi)

// Adding new Annotation

anew.setPathClass(aClass)

anew.setLocked(true)

tumorShrinked.add(anew)

}

removeObjects(getAnnotationObjects().findAll{it.getPathClass() == getPathClass("Tumor")}, true)

hierarchy.insertPathObjects(tumorShrinked)

print("Shrinked Tumor annotations created")

// Create Stroma TN and Stroma TD annotations

//**

def radiusMicronsTN = 50.0

double expandPixelsTN = radiusMicronsTN / pixelSize

def cores = hierarchy.getTMAGrid().getTMACoreList()

for (c in cores){

if (cores.indexOf(c)% 10 == 0){

print("Processing Tumor Neighborhood annotations for core nr. " + (cores.indexOf(c)+1))

}

def annotationsToAdd = []

// Instanciate PathObjects, ROIs, Geometries

PathObject annotationTissue

PathObject annotationTumor

PathObject annotationTN

PathObject annotationTD

//ROI roiStroma

//ROI roiTumor

//ROI roiTN

//ROI roiTD

Geometry geomTissue

Geometry geomTumor

Geometry geomTN

Geometry geomTD

def plane

// Cores with NO tissue annotation

if (c.getDescendantObjects().findAll{it.getPathClass() == tissue && it.isAnnotation() == true} == []){

print("Core without Tissue found: " + c)

continue

}

// Cores with tissue AND tumor annotations

else if ((c.getDescendantObjects().findAll{it.getPathClass() == tumor && it.isAnnotation() == true} != []) && (c.getDescendantObjects().findAll{it.getPathClass() == tissue && it.isAnnotation() == true} != [])) {

// Create Stroma TN annotation

// Creating PathObjects

annotationTissue = c.getDescendantObjects().findAll{it.getPathClass() == tissue && it.isAnnotation() == true}.get(0)

annotationTumor = c.getDescendantObjects().findAll{it.getPathClass() == tumor && it.isAnnotation() == true}.get(0)

// Creating ROIs

def roiTissue = annotationTissue.getROI()

def roiTumor = annotationTumor.getROI()

// Creating Geometries

geomTissue = roiTissue.getGeometry()

geomTumor = roiTumor.getGeometry()

plane = roiTumor.getImagePlane()

// Creating new Geometries and Stroma TN Annotations

geomTN = geomTumor.buffer(expandPixelsTN)

geomTN = geomTN.difference(geomTumor)

geomTN = geomTN.intersection(geomTissue)

geomTN = GeometryTools.homogenizeGeometryCollection(geomTN)

def roiTN = GeometryTools.geometryToROI(geomTN, plane)

annotationTN = PathObjects.createAnnotationObject(roiTN)

annotationTN.setPathClass(stromaTN)

annotationTN.setLocked(true)

if (roiTN.getArea() > 0){

annotationsToAdd.add(annotationTN)

}

// Creating new Geometries and Stroma TD Annotations

geomTD = geomTissue.difference(geomTumor)

geomTD = geomTD.difference(geomTN)

geomTD = GeometryTools.homogenizeGeometryCollection(geomTD)

def roiTD = GeometryTools.geometryToROI(geomTD, plane)

annotationTD = PathObjects.createAnnotationObject(roiTD)

annotationTD.setPathClass(stromaTD)

annotationTD.setLocked(true)

if (roiTD.getArea() > 0){

annotationsToAdd.add(annotationTD)

}

// Deleteing Tissue annotation

removeObject(annotationTissue, true)

print("TN and TD created")

}

// Cores with ONLY Tissue annotations (without tumor)

else if ((c.getDescendantObjects().findAll{it.getPathClass() == tumor && it.isAnnotation() == true} == []) && (c.getDescendantObjects().findAll{it.getPathClass() == tissue && it.isAnnotation() == true} != [])) {

annotationTissue = c.getDescendantObjects().findAll{it.getPathClass() == tissue && it.isAnnotation() == true}.get(0)

annotationTD = annotationTissue

annotationTD.setPathClass(stromaTD)

annotationTD.setLocked(true)

annotationsToAdd.add(annotationTD)

// Deleteing Tissue annotation

removeObject(annotationTissue, true)

print("Only TD created")

}

// Adding 3 Annotations into core hierarchy(Stroma TN, Stroma TD, Stroma TI)

// Using this method will sort hierarchy already! No need for resolveHierarchy() afterwards

if (annotationsToAdd != null){

hierarchy.insertPathObjects(annotationsToAdd)

print(c)

print(annotationsToAdd)

}

}

print("Spatial annotations created for " + cores.size() + " cores!")

// Removing parentless Annotations (= Polylines without area +- Tumor?)

def annotationsToRemove = getAnnotationObjects().findAll{it.getLevel() == 1}

print("Removing parentles annotations: " + annotationsToRemove)

removeObjects(annotationsToRemove, true)

// Allocate cells according to PathClass

for (cell in getDetectionObjects()) {

def CD3 = measurement(cell, "CD3")

def CD8 = measurement(cell, "CD8")

// Classify tumor cells

if ((cell.getParent().getPathClass() == tumor) && (CD3 == 0) && (CD8 == 0)) {

cell.setPathClass(tumor)

}

// Classify negative stroma cells

else if ((CD3 == 0) && (CD8 == 0)) {

cell.setPathClass(stromaNeg)

}

// Classify CD3 stroma cells

else if ((CD3 == 1) && (CD8 == 0)) {

cell.setPathClass(stromaCD3)

}

// Classify CD8 stroma cells

else if ((CD8 == 1)) {

cell.setPathClass(stromaCD8)

}

}

// Create measurements for annotations

Set classList = []

for (object in getAllObjects().findAll{it.isDetection()}) {

classList << object.getPathClass()

}

for (annotation in getAnnotationObjects()){

def totalCells = []

def ml = annotation.getMeasurementList()

double annotationArea = annotation.getROI().getArea() * pixelSize * pixelSize / 1000000

totalCells = annotation.getDescendantObjects().findAll{it.isDetection() == true}

ml.putMeasurement("Area mm^2", annotationArea)

ml.putMeasurement("Total cells", totalCells.size())

for (aClass in classList){

if (aClass){

if (totalCells.size() > 0){

def cells = totalCells.findAll{it.getPathClass() == aClass}

ml.putMeasurement(aClass.getName()+" cells Num", cells.size())

ml.putMeasurement(aClass.getName()+" cells %", cells.size()*100/totalCells.size())

ml.putMeasurement(aClass.getName()+" cells /mm^2", cells.size() / annotationArea)

ml.close()

}

else {

ml.putMeasurement(aClass.getName()+" Num cells", 0)

ml.putMeasurement(aClass.getName()+" %", 0)

ml.putMeasurement(aClass.getName()+" cells /mm^2", 0)

ml.close()

}

}

}

}

print("Annotation measurements calculated!")

// Edit TMA measurement list

for (c in cores){

def ml = c.getMeasurementList()

for (annotation in c.getChildObjects().findAll{it.isAnnotation() == true}){

ml.putMeasurement(annotation.getPathClass().toString() + ": Total cells", measurement(annotation, "Total cells"))

ml.putMeasurement(annotation.getPathClass().toString() + ": Area mm^2", measurement(annotation, "Area mm^2"))

for (aClass in classList){

String classNum = aClass.getName().toString() + " cells Num"

ml.putMeasurement(annotation.getPathClass().toString() + ": " + aClass.getName() + " cells Num", measurement(annotation, classNum))

String classPercent = aClass.getName().toString() + " cells %"

ml.putMeasurement(annotation.getPathClass().toString() + ": " + aClass.getName() + " cells %", measurement(annotation, classPercent))

String classDensity = aClass.getName().toString() + " cells /mm^2"

ml.putMeasurement(annotation.getPathClass().toString() + ": " + aClass.getName() + " cells /mm^2", measurement(annotation, classDensity))

}

}

ml.close()

}

print("TMA measurements created!")

// Export TMA Measurements

saveTMAMeasurements("D:/Validation cohort/Results")

print("TMA measurements exported!")
